# Micro-computed tomography for the identification and characterization of archaeological lime bark

**DOI:** 10.1038/s41598-023-33633-x

**Published:** 2023-04-20

**Authors:** Jörg Stelzner, Sebastian Million, Ingrid Stelzner, Oliver Nelle, Johanna Banck-Burgess

**Affiliations:** 1 Leibniz-Zentrum für Archäologie, Ludwig-Lindenschmit-Forum 1, 55116 Mainz, Germany; 2 Landesamt für Denkmalpflege im Regierungspräsidium Stuttgart, Fischersteig 9, 78343 Gaienhofen-Hemmenhofen, Germany; 3 Landesamt für Denkmalpflege im Regierungspräsidium Stuttgart, Berliner Straße 12, 73728 Esslingen am Neckar, Germany

**Keywords:** Plant physiology, Characterization and analytical techniques, Imaging techniques, Microscopy

## Abstract

In the Neolithic pile-dwelling settlements of southwestern Germany, bark played a prominent role in the production of technical textiles. So far, the inner bark (phloem) of the lime tree (genus *Tilia*) could be detected most frequently. Microscopic examination of anatomical features can determine the taxon, requiring manipulation of samples and archaeological objects. In this study, micro-computed tomography (µCT) was reviewed as a method for determining the woody taxon and obtaining additional information from the inner bark. To this end, modern bark samples from different tree organs of lime were first analysed using both µCT and transmitted light microscopy. Both methods were able to detect all characteristic anatomical features in the phloem and identify the genus. With analysis based on µCT data, further anatomical information can be obtained. For example, the shape of the phloem rays in the bast strips can provide information on the position within the bark and on the original organ diameter. These results obtained on modern material were verified on four samples from archaeological objects. Based on µCT, all samples could be clearly identified as lime and in two cases conclusions could also be drawn about the raw material. This approach could lead to new results and interpretations in archaeological sciences.

## Introduction

**Figure 1:**
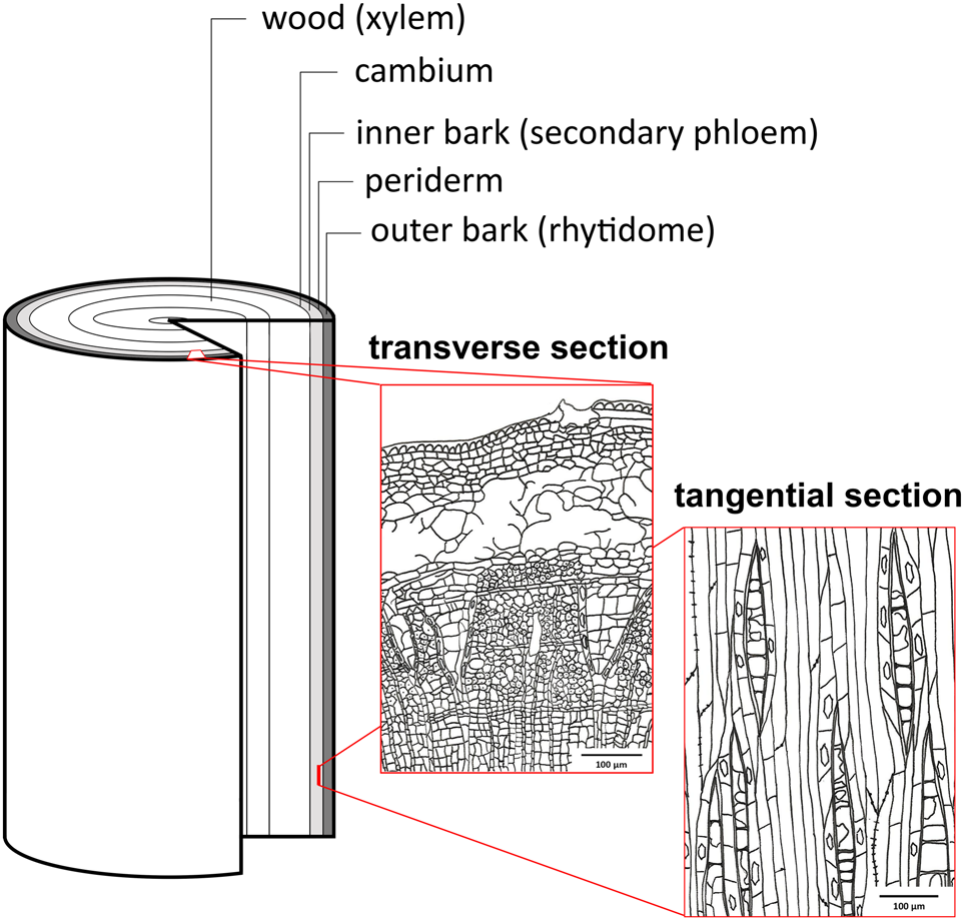
Schematic illustration of the bark and two exemplary drawings from transverse and tangential section of *Tilia*.

Tree bast was used to make the earliest textiles in human history in Europe, which have been excavated so far^1^. Through cell division, the vascular cambium builds up xylem cells (wood) towards the pith and phloem cells (tree bast, inner bark) towards the outside of the trunk during the thickening growth of the tree trunk (Fig. 1). For example, in the pile-dwelling settlements of southwestern Germany, tree bast played a prominent role. This is especially true for the production of technical textiles, where functional properties are relevant, while aesthetics and decorative character are not^2^. Thanks to the exceptional preservation conditions for organic remains in prehistoric wetland settlements, a number of textiles and bark containers survived that must have been indispensable in the daily working life of the population^3^. The anaerobic conditions of lake sediments preserved strings, nets, lightweight and unbreakable collection containers, baskets, and mats. They represent only a fraction of those textile objects to which primarily a practical function could be attributed.

Within the research project “Textile Handicraft in the Prehistoric Wetland Settlements at Lake Constance and in Upper Swabia—Requirements for Textiles and their Perception” (THEFBO) it is investigated which skills were present in the selection, preparation and processing of raw materials during the period under investigation. A necessary prerequisite for the evaluation of archaeological finds is the material determination. The identification of certain materials is especially required for a better understanding of the technological capabilities of early humans. Usually, the different types of bast are identified by characteristic anatomical features under the microscope. So far, the bast of lime (*Tilia*), oak (*Quercus*), willow (*Salix*), elm (*Ulmus*), and poplar (*Populus*) has been identified on archaeological objects. Among the tree bast used in prehistoric Central and Southern Europe, both the bast and the bark of lime play a prominent role^4–10^.

Identifying the genus on the basis of anatomical features in artifacts made of bark from archaeological collections is usually difficult. For example, influences from the preparation of the fibres during the manufacturing processes, repeated use of the manufactured objects, or charring by fire can significantly alter the condition of the materials. Relevant anatomical features may also be affected by degradation during thousands of years of storage in the ground or obscured by adhering sediments, decomposition products and conservation agents^11,12^. For ethical reasons, destructive sampling to obtain cross-sections may not be justifiable for smaller finds of great importance. Even if it were acceptable, cutting cross-sections from archaeological material can be extremely difficult or impossible due to its condition. It may also be necessary to take and prepare multiple samples to make a reliable determination, as features may not be readily apparent due to the above circumstances. However, this only becomes apparent when time and resources are invested in cutting and preparing the specimen.

Among the non-destructive methods currently available, X-ray computed tomography (CT) is a powerful imaging technique that allows to inspect the entire volume of the sample at different spatial resolutions^13^. For the anatomical determination of plants, bark and wood a high resolution is required. This can be achieved, for example, with CT using synchrotron radiation^14,15^ or industrial micro-computed tomography (μCT). Critical to the success of archaeological wood and bark analysis with µCT is the resolution of the measurement. The resolution depends on the sample size and can be for small samples (< 1 mm) at a voxel size of < 1 µm. The µCT provides therefore sufficient resolution for small samples to determine wood^16,17^ or plants^18^ based on their characteristics. The same is applicable to archaeological wood samples^19^. Determination of wood species of archaeological finds was also possible when they were present in a mineralized state on iron objects^20,21^ or as highly decomposed wood fragments in a block of soil^21^. Using µCT, the internal structures and mineral phases of charcoal^22^ and charred archaeological parenchyma from Papua New Guinea^23^ were also investigated. µCT was also used to examine morphological characteristics of archaeological plant remains^24^ and to analyse plant material from archaeological basketry^25^. A previous study^26^ examined recent bark samples using µCT. Here, the identification of diagnostic features of lime, willow, and oak was successful—The genera of wood which were predominantly identified in stone-age textiles^1,6,7^. A distinction between the mid-european species small-leaved lime (*Tilia cordata*) and large-leaved lime (*Tilia platyphyllos*) is not possible on the basis of the anatomical features neither with the µCT nor with the conventional microscopy, because of similar anatomy. This is also true for *Tilia tomentosa*, which is outside the here considered areal, being naturally distributed in southeast Europe. In this study, it will be examined whether these observations on modern material can also be used for the investigation of archaeological finds. Since all the objects examined here were also made of lime bark, the investigation is limited to the genus *Tilia*. The three-dimensional representation of the anatomical features of the bark in the µCT images could help to answer questions about the quality of the raw material used. It was also verified whether it was possible to distinguish the organ from which the material originated. Whether this is the trunk or a branch is an interesting aspect to understand the manufacturing techniques.

## Materials and methods

### Modern lime bark

The genus of bark material is usually identified analogously to that of wood by determining the characteristic anatomical features under the microscope. Two main directions of cutting are examined for bark: transverse and tangential. For the identification of archaeological objects, the most important features are analysed using transmitted light microscopy with a magnification of 50x to 500x^4,7,8,27^. The modern samples were taken from a twig and a shoot of small-leaved lime and from a trunk and a twig of large-leaved lime. The diameter of all measured samples is 5 mm (Table 1). The samples 1 and 3 were taken with a 5 mm increment borer (Haglöf Sweden). The samples 2 and 4 are split in quarters of a round shoot and twig, respectively.

**Table 1:** Samples from recent lime bark.

Sample	No	Species	Sampling site	Plant organ	Organ diameter	Age	Dimensions sample
1	TIL 1	Small-leaved lime *(Tilia cordata)*	Hemmenhofen	Branch	85 mm	38	L.: 10 mm, Ø: 5 mm
2	TIL 3		Moos	Shoot	8 mm	1	L.: 4 mm, Ø: 5 mm
3	TIL 12	Large-leaved lime *(Tilia platyphyllos)*	Radolfzell	Trunk	280 mm	Unknown	L.: 10 mm, Ø: 5 mm
4	TIL 11.2		Radolfzell	Twig	9 mm	3	L.: 6 mm, Ø: 5 mm

### Archaeological objects

**Figure 2:**
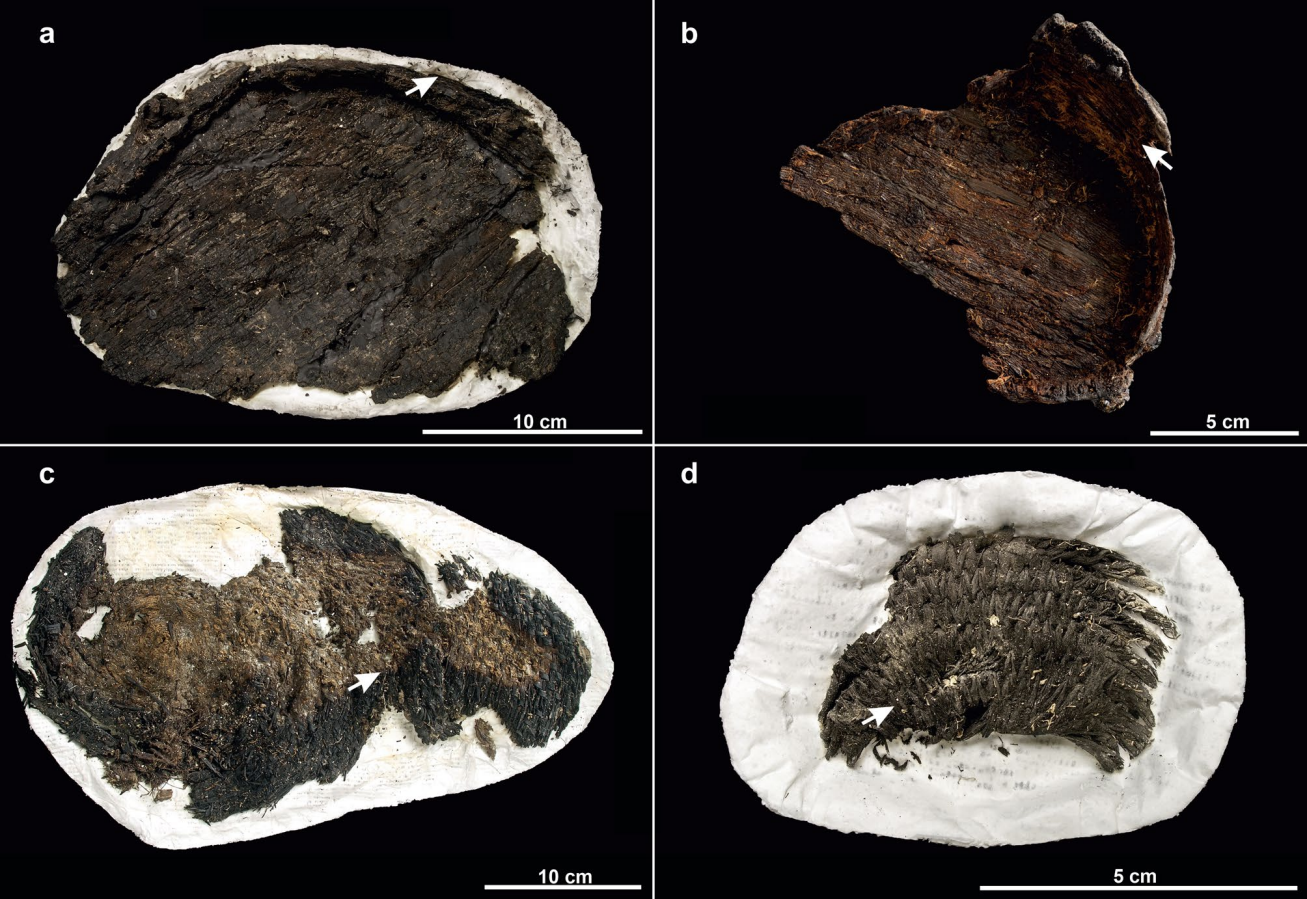
Archaeological objects: (**a**) bark container (sample 5), (**b**) bark container (sample 6), (**c**) coiled basket (sample 7), (**d**) coiled basket (sample 8). The arrows indicate the locations of sampling. Photos: (**a**) Matthias Hoffmann/ALM, (**b**) Yvonne Mühleis/LAD, (**c**,**d**) Sebastian Böhm/FAU.

The archaeological lime bark samples were taken from four different objects (Fig. 2). The finds came from lake-shore settlements at Lake Constance: Hornstaad-Hörnle and Sipplingen-Osthafen (Table 2). Both are multi-phase Neolithic settlements on the shore of lake Constance^28–31^. Samples 7 an 8 derived from coiled baskets made of bundles of steams^6^. The coils were sewn together with so-called binders made of fine bark strips. The samples 5 and 6 were taken from cylindrical bark container in which the walls and bottom were sewn together with strips of bast. The objects are not (sample 5, 6), entirely (sample 8) or partly charred (sample 7). The conserved objects were treated with an aqueous solution of 8% polyethylene glycol 400 and 5% Luviskol K30 before freeze-drying^32^.

**Table 2:** Samples from archaeological objects.

Sample	Find-no	Archaeological site	Object	Function	Condition	Dimensions sample
5	Ho85 52/50-20C	Hornstaad-Hörnle	Bark container	Side	Not conserved	L.: 2 mm, Ø: 4 mm
6	Ho87 42/37-17	Hornstaad-Hörnle	Bark container	Side	Conserved	L.: 2 mm, Ø: 5 mm
7	Ho90 62/58-34	Hornstaad-Hörnle	Coiled basket	Binder	Charred, conserved	L.: 1 mm, Ø: 4 mm
8	Si83 125-1006	Sipplingen Osthafen	Coiled basket	Binder	Charred	L.: 1 mm, Ø: 4 mm

### Methods

The examination of the samples was done at the Research Institute for Precious Metals and Metal Chemistry (FEM), Schwäbisch Gmünd, Germany, using a GE Phoenix nanotom m® X-ray CT system with 180 kV microfocus tube and 3072 × 2400-pixel dxr-500L1 flat panel detector. The measurements were performed with a voltage of 140 kV and a current of 28 µA. The integration time was 1500 ms and 1000 projections were recorded. The resolution of the data is 5 µm voxel edge length. The analysis and visualisation of the CT data was performed with the software VGStudio MAX 3.4®.

The results obtained from the µCT on the modern samples were verified by comparing them with the results of the microscopic wood species determination which was performed on the same samples. A sledge microtome (WSL Lab-Microtome) was used to prepare the anatomical thin sections^33^. Depending on the species or cutting direction, the thickness of the thin sections varied between 10 and 25 µm. These thin sections were stained in a solution of safranin and astra blue (1:1)^34^. The microscopic structure was observed using a Nikon Eclipse LV 100 ND transmitted light microscope at magnifications between 50x and 500x with polarised light. Images from the Nikon microscope were captured using a Nikon DS-Ri2 camera and Nikon’s NIS-Elements 4.50 software at a resolution of 4908 × 3264 pixels.

## Results and discussion

### Features detected with µCT on modern samples

#### Comparison of features detected with µCT and microscopy

**Figure 3:**
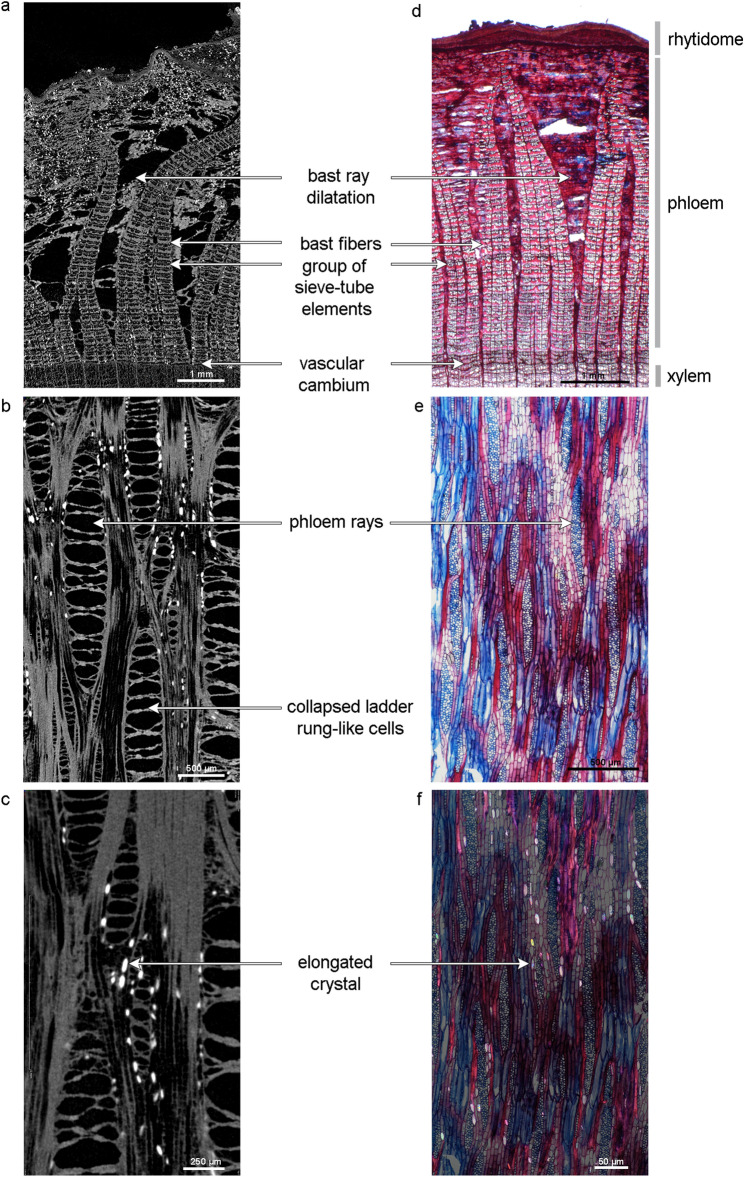
Characteristics of lime (sample 1, Table 1) recorded with µCT (left) and microscopy (right) with transmitted (**d**,**e**) and polarised (**f**) light of thin sections stained to highlight lignified (red) and non-lignified (blue) cells. (**a**) and (**d**) represent the transversal, and (**b**), (**c**), (**e**), (**f**) the tangential section. The tangential sections in (**b**) and (**c**) show characteristic ladder-like-structures that are build when the bark dries out. In pictures (**e**) and (**f**), on the other hand, the cell structure is still intact.

The characteristic anatomical features of the bark are visible in both the microscopic images and the µCT data (Fig. 3). Thus, the features necessary for identification of all lime samples can be seen in the transverse and tangential sections. In the cross-section of sample 1 (Fig. 3a,d), the primary phloem rays widen towards the rhytidome, and this frequently substantial dilatation is shaped like a triangular wedge^35^. In addition, the tangential rows of bast fibres are clearly visible in both images, as is the lime-typical pattern of phloem fibres that run laterally to the vascular cambium, thus surrounding it on three sides. In a tangential section (Fig. 3b,e), the phloem rays have a spindle shape^36^, and are widening towards the outer bark (dilatation) as it can be observed in the cross section. When they dry out, the phloem ray cells collapse to form a characteristic striped structure (Fig. 3b). Druse crystals are found in the bast rays, mostly in the dilatation zones (Fig. 3c,f). The elongated (large and hexagonal) calcium oxalate crystals in the parenchyma, which are also clearly visible in the tangential section, are typical of *Tilia*. They look rectangular in the transverse plane and are present only near the phloem rays. Sclereids in the shape of stone cells are absent^4,7,35,37^.

#### Additional visualisation options for the µCT data

**Figure 4:**
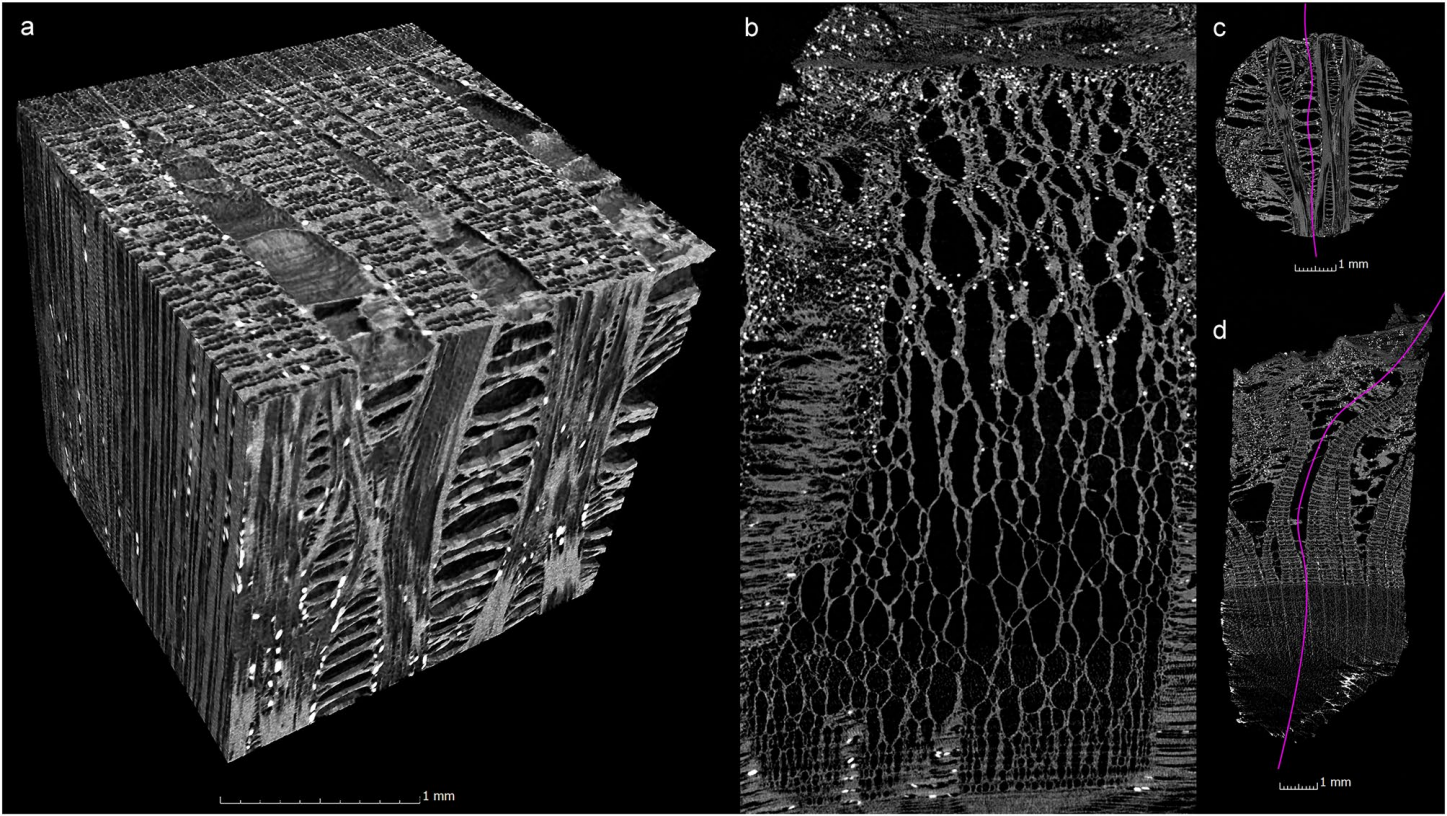
Three-dimensional µCT representation (**a**) and radial section along a phloem ray (**b**) in the bark of lime (sample 1). The radial plane of view of the phloem ray (**b**) is illustrated by the pink line in the tangential (**c**) and transversal (**d**) sections.

**Figure 5:**
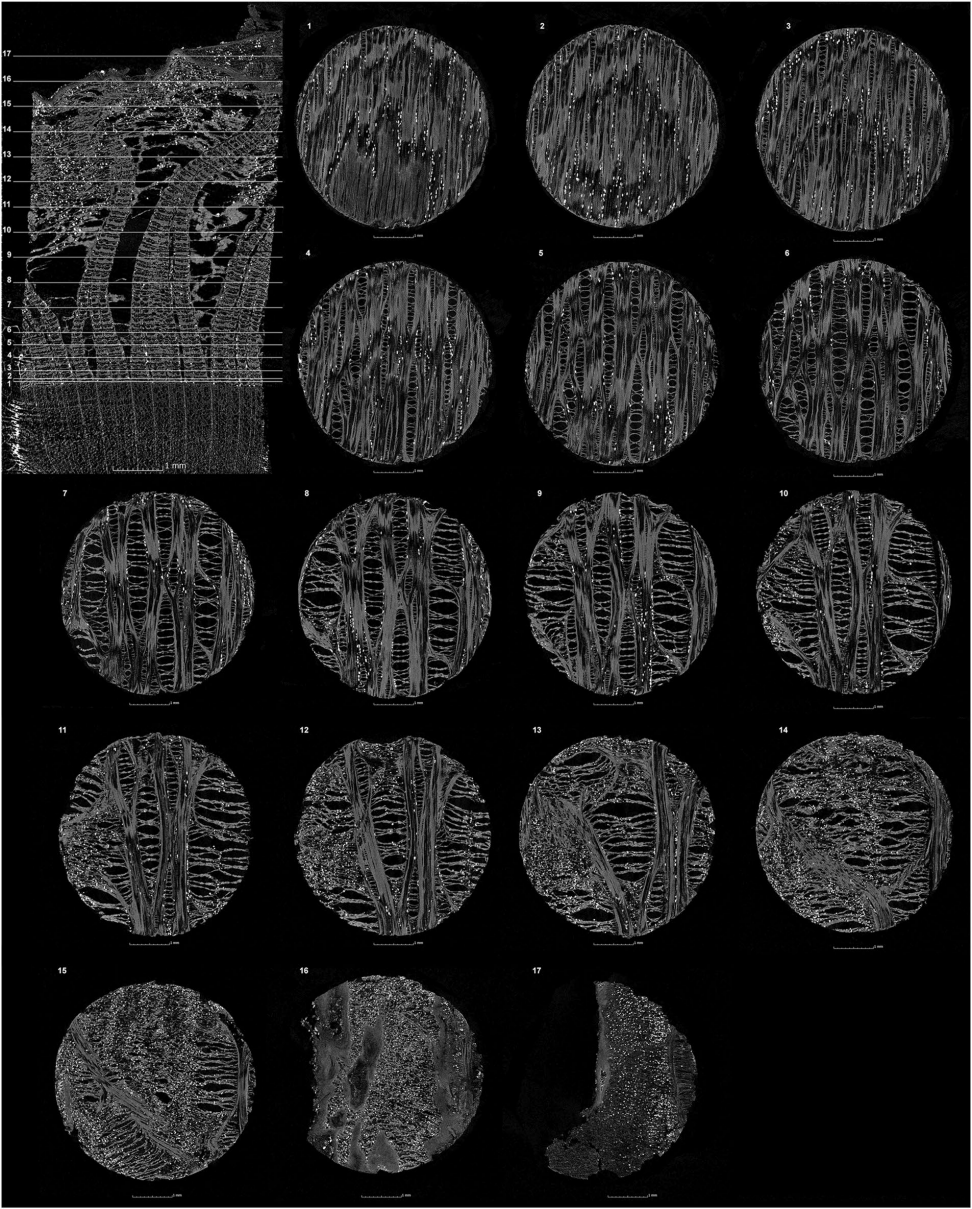
µCT transversal (upper left) and tangential sections of lime (sample 1). The 17 tangential sections are presented with their position on the transversal section.

The µCT data also allow a detailed three-dimensional representation of the phloem beyond the display of the individual sectional images (Figs. 4, 5). The anatomical structure of the bark of the different tree species is demonstrated by visualising the specimens in all three sectional planes (transverse, radial, tangential). The features previously described in the cross sections are also visible in the three-dimensional visualisation.

Compared to microscopy, the three-dimensional data of µCT offers additional possibilities for visualising the anatomy of the phloem. For example, any number of slices can be taken at any location, making it possible to track and document the progression of anatomical features such as phloem rays. In this way, changes in the anatomy of the phloem can also be tracked across the entire sample. Figure 5 illustrates how the phloem rays of small-leaved lime (sample 1) increase in height and width from the cambium to the outer bark. Also, on the 20 mm^2^ area of the increment core, the number of phloem rays decreases from in the comparable area c. 25 near the cambium (Fig. 5.3) to approximately 3 near the rhytidome (Fig. 5.13). Apparently, phloem rays can separate or merge during their radial course. The processing of the µCT data also allows sections to be created over several planes, which makes it possible to display the entire winding course of a phloem ray in the radial plane (Fig. 4b–d).

#### Analysis of anatomical dimensions in µCT data

In the µCT data, it is possible to measure the sizes and angles of the different anatomical features. Thus, in the case of lime bark, these measurements can be used to make statements about the radial position of the bast layer in the phloem: In the outer region of the phloem, the rays are magnified due to phloem ray dilation. Some phloem rays dilate towards the outer bark^7^. In the case of the small-leaved lime examined here (sample 1), the phloem rays are 0.05 mm wide at the cambium, and are widening to 3.5 mm towards the rhytidome, which is also shown in Fig. 5. In the examined large-leaved lime (sample 3), 0.04 to 1.8 mm wide phloem rays could be measured in each case (Table 3). The radial representation of the phloem ray of the small-leaved lime illustrates the increasing width of the phloem rays towards the outer bark (Fig. 4b).

**Table 3:** Measurements of anatomical features extracted from µCT data.

Sample	Diameter organ in mm	Bark thickness in mm	Phloem rays			
			Width in mm		Angle in °	
			Min	Max	Min	Max
1	–	7.6	0.05	3.50	12	22
2	7.3	0.7	0.03	0.90	12	90
3	–	7.5	0.04	1.80	10	30
4	9.5	1.1	0.04	0.95	12	70
5	22.9	2.9	0.03	1.90	12	55
6	–	2.5	0.04	0.90	13	45
7	–	1.0	0.04	0.30	10	50
8	–	0.8	0.03	0.35	10	50

Diameter and age of the plant are determining factors for the thickness of the bark^38^. The measurements of the bark thickness of the studied samples in the µCT data confirm this. Thus, the bark of the twig and shoot is significantly thinner than the bark of the trunk or branch (Tables 1, 3). The age of the plant organ cannot be estimated by using the thickness of the bark, rather, the number of tangential rows of fibres provides a more efficient approach. Further research is definitely necessary concerning this aspect.

**Figure 6:**
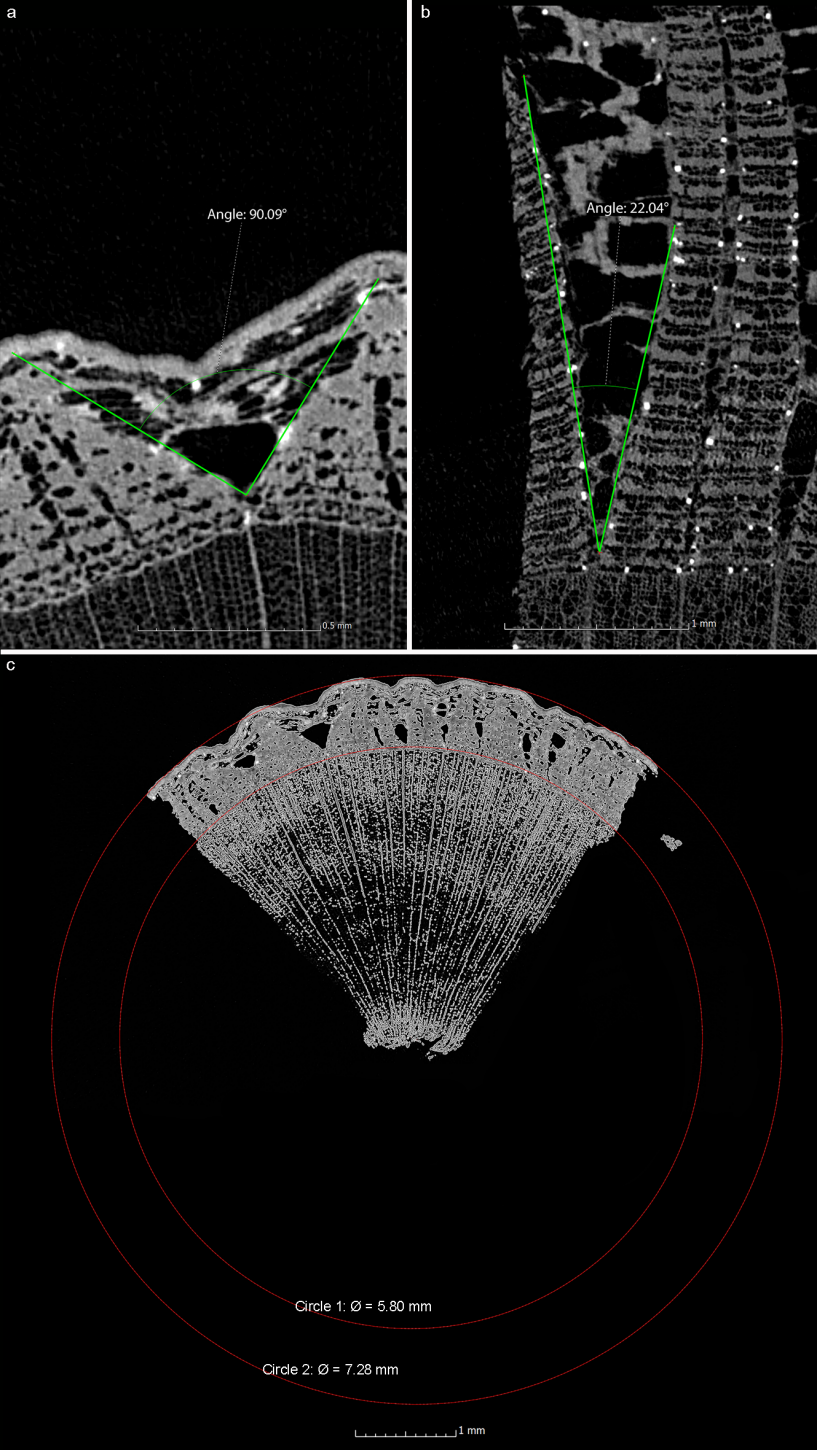
µCT cross sections of the largest angle of a phloem ray in the bark of lime branch (**a**) sample 2 and (**b**) sample 1. (**c**) µCT cross section showing the diameter of the organ from which the bark of a lime shoot (sample 2) was taken, calculated by extrapolating the complete circumference and tree-rings from the existing sample.

The shape of the phloem rays in these samples also provides information about the origin of the bark, which mainly concerns the diameter of the branch or trunk. Phloem rays in bast layers originating from an organ with a smaller diameter form a larger angle. The curvature on the outside of the bark depends on the circumference or diameter of the tree organ in question. Consequently, the dilating phloem rays must compensate for greater circumferential growth on the outside of the bark for smaller diameters than they do for larger diameters. Accordingly, the diameter and roundness of the outer bark diameter and the angles of the phloem rays will vary with the age of the tree organ. Measurements of the angles on our samples confirm this, with the angle on a branch and trunk being much smaller than on a shoot or branch (Tables 1, 3, Fig. 6a,b).

The evaluation of the curvature in the sample gives information about the diameter of the organ, which can be calculated from the µCT data (Fig. 6c). This is only possible for the smaller diameters of organs, as the curvature is hardly measurable for larger diameters due to the small sample size, which would lead to strong fluctuations. The diameters of 7.3 and 9.5 mm determined on samples 2 and 4 are very close to the diameters of the source material, which are given as 8 and 9 mm (Tables 1, 3).

### Features detected with µCT on archaeological samples

#### Anatomical features detected with µCT

**Figure 7:**
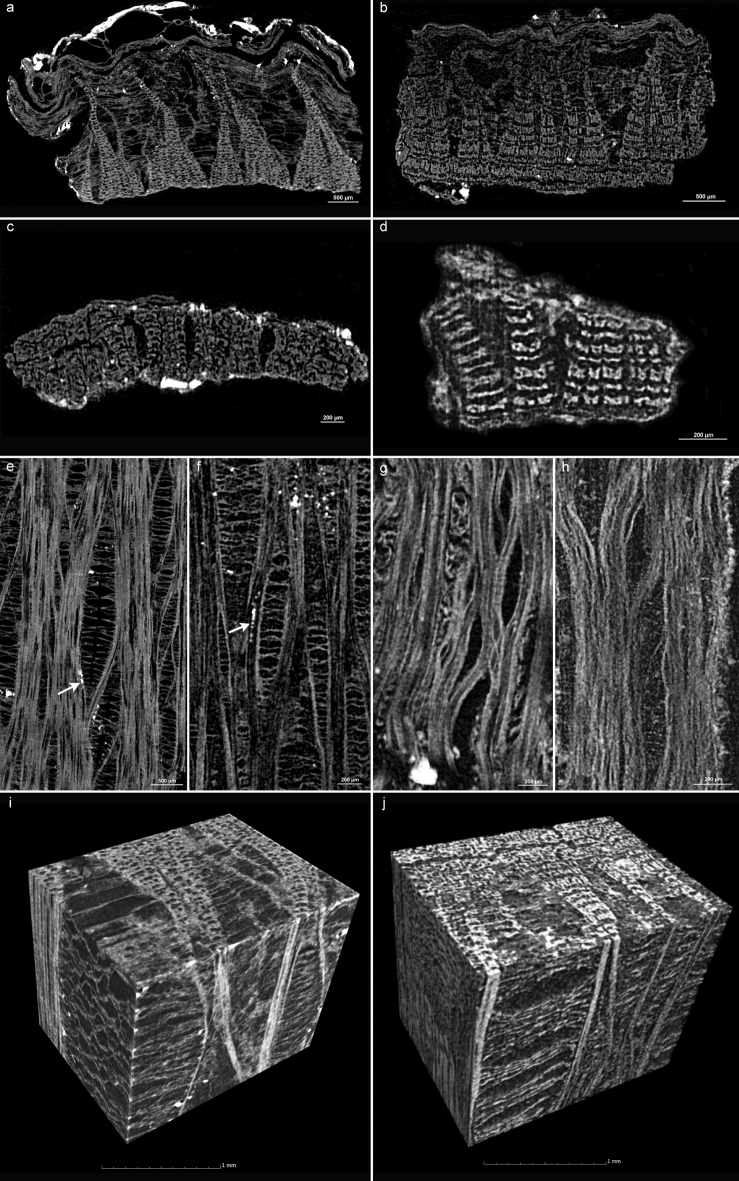
Characteristics of lime in the µCT data of the archaeological samples (Table 1b) in the cross sections of the sample 5 (**a**), 6 (**b**), 7 (**c**) and 8 (**d**) and the tangential section of sample 5 (**e**), 6 (**f**), 7 (**g**) and 8 (**h**). The arrows indicate the elongated crystals. Three-dimensional µCT representations of sample 5 (**i**) and 6 (**j**).

The characteristic anatomical features observed in the microscopic images and in the µCT data of the modern lime samples (Fig. 3) are also found in the µCT data of the archaeological samples (Table 2). Both, the phloem ray dilatation in the cross-section (Fig. 7a–d), as well as the extremely wide phloem ray dilatation (Fig. 7e,f) in the tangential section are visible in the µCT data. In the samples 5 and 6 of the bark containers, the features are slightly more visible than in samples 7 and 8 of the binder of the coiled baskets. In these samples, the phloem rays are somewhat crushed (Fig. 7c,d,g,h). In addition, in contrast to these samples, in samples 5 and 6, remnants of elongated calcium oxalate crystals in the parenchyma appear to be visible in the tangential sections (Fig. 7e,f). The section directions of these samples can also be well visualized three-dimensionally (Fig. 7i,j).

#### Analysis of anatomical dimensions in µCT data

The sizes and angles of the different anatomical features of the archaeological lime bark samples were measured in the µCT data (Table 3). It was possible to measure the thickness of the bark in the samples 5 and 6 and of the phloem in the samples 7 and 8, respectively. Additionally, the widening and the minimum and maximum angles of the phloem rays could be measured. The interpretation of the measured values of the archaeological samples is difficult, because especially the samples of the binder of the coiled baskets (sample 7 & 8) are deformed and they do not represent the whole bark. Since the vertex of the phloem rays is present, this could also be explained by the fact that only the part near to the cambium of the bark was used for the binders here.

The lime bark of sample 5 was the only archaeological sample where the condition of the sample allowed to obtain information about the diameter of the organ. The other samples were too small or too deformed for this purpose and an original rounding was not traceable here. This seems to be difficult with archaeological samples anyway due to the long storage and the original processing and use of the material. In the case of sample 5, the determined values would nevertheless fit very well to the assumed correlation of the maximum angle of the phloem rays to the organ diameter. In relation to the recent samples (Tables 1, 3) with large organ diameters and narrow phloem ray angles (sample 1 & 3) and those with small organ diameters and large phloem ray angles (sample 2 & 4), the archaeological sample occupied a value in the middle with an organ diameter of 22.9 mm and a maximum phloem ray angle of 55°. This mean value is also confirmed by the bark thickness of 2.9 mm.

In the case of sample 6, the narrower maximum angle of the phloem ray of 45° indicates a larger organ diameter than sample 5. In contrast, the bark of sample 6 is slightly narrower than that of sample 5, at 2.5 mm.

## Conclusion

The taxonomic identification of modern lime bark based on µCT was demonstrably successful. The decisive features for genus determination could be made visible in the cross-sectional and tangential images. One limitation of µCT is that this method cannot serve with an answer with the differentiation of cell types. Separating cellulose and lignin rich cells is only possible with staining, in our case with Astrablue and Safranine. In contrast, µCT allows the anatomy to be represented three-dimensionally without alterations (i.e. not idealized), which would not be the case with model drawings. This allows the inhomogeneous material to be captured more objectively. In addition, the technique allows refinement of the anatomical study of the lengths and widths of the individual elements in “virtual sections” of the entire sample. Moreover, µCT can also be used to obtain information that cannot be recorded with conventional microscopic analysis. For example, the changes in the phloem rays from the cambium to the bark can be documented in detail, and the radial position can be estimated more accurately. Due to the dilatation of phloem rays towards the outside of the bark, the bast quality when harvesting the bark for the production of textiles (e.g. cords, strings) from this zone is coarser compared to the material derived from near the vascular cambium. Here, the phloem rays appear high and very narrow in the tangential plane, resulting in a very fine morphology of the bast layers in this location. This allows it to draw conclusions with regard to the quality of the bast used for processing. Textile research, however, does not confine itself to pursuing questions about the quality of the material used, the age or the dimensions of the examined source material are also of interest. Depending of the archaeological find, it appears that different plant organs of a specific diameter or circumference were intentionally selected. µCT also allows the different anatomical features of the whole samples to be measured, which in turn allows a better comparison of the respective maximum dimensions of these features. For example, the material thickness of the bark or the angles of the phloem rays can thus be an indication of the original diameter or the specific organ of the material used. Measuring the different sizes of the phloem rays gives an indication of the diameter of the sample, which for smaller branches or twigs can only be determined from the samples themselves.

First investigations on archaeological bark objects show here that a determination based on the anatomical features in lime bast is possible with µCT. In addition, information on the source material could also be obtained in individual cases with a corresponding state of preservation. The information that can be obtained from the method about the material used allows conclusions to be drawn about the type, age and quality of the raw material selected at that time. Consequently, the µCT technique also offers promising possibilities for analysing archaeological objects. In the future, an examination of a larger number of objects could lead to further insights with regard to the knowledge and skills of early people in the manufacturing of textiles for different purposes.

The application of µCT also enables the analysis of smaller fragments of a particular material, which can be done with little or no preparation of the sample. Furthermore, the entire sample can be visualised three-dimensional, and features can be captured in cross-sectional images at any point in the sample without the need to destroy the sample. This is particularly important for archaeological samples, which can theoretically be reintegrated into the sampled object at a later date.

## Data Availability

The datasets generated during and/or analysed during the current study are available from the corresponding author on reasonable request.
