# Author Correction: Micro-computed tomography for the identification and characterization of archaeological lime bark

**DOI:** 10.1038/s41598-023-37591-2

**Published:** 2023-06-28

**Authors:** Jörg Stelzner, Sebastian Million, Ingrid Stelzner, Oliver Nelle, Johanna Banck-Burgess

**Affiliations:** 1 Leibniz-Zentrum für Archäologie, Ludwig-Lindenschmit-Forum 1, 55116 Mainz, Germany; 2 Landesamt für Denkmalpfege im Regierungspräsidium Stuttgart, Fischersteig 9, 78343 Gaienhofen-Hemmenhofen, Germany; 3 Landesamt für Denkmalpfege im Regierungspräsidium Stuttgart, Berliner Straße 12, 73728 Esslingen am Neckar, Germany

Correction to: *Scientific Reports* 10.1038/s41598-023-33633-x, published online 20 April 2023

The Author Contributions section in the original version of this article contained an error, where the author Oliver Nelle was incorrectly identified as ‘O.L.’

“J.S., S.M. and I.S. designed, analysed and interpreted the data and wrote the manuscript. O.L. and J.B. supported the analysis of the data as well as contributed to the manuscript writing. All authors read and approved the final manuscript.”

now reads:

“J.S., S.M. and I.S. designed, analysed and interpreted the data and wrote the manuscript. O.N. and J.B. supported the analysis of the data as well as contributed to the manuscript writing. All authors read and approved the final manuscript.”

The original Article has been corrected.

